# Bcl-2 expression predicts sensitivity to chemotherapy in breast cancer: a systematic review and meta-analysis

**DOI:** 10.1186/1756-9966-32-105

**Published:** 2013-12-27

**Authors:** Dong Yang, Min-Bin Chen, Li-Qiang Wang, Lan Yang, Chao-Ying Liu, Pei-Hua Lu

**Affiliations:** 1 Department of General Surgery, Kunshan First People’s Hospital Affiliated to Jiangsu University, 91 Qianjin Road, Kunshan, Jiangsu Province 215300, China; 2 Department of Medical Oncology, Kunshan First People’s Hospital Affiliated to Jiangsu University, 91 Qianjin Road, Kunshan, Jiangsu Province 215300, China; 3 Department of Breast Surgery, The Third Affiliated Hospital of Soochow University, 185 Juqian Street, Changzhou, Jiangsu Province 213000, China; 4 Department of Medical Oncology, Wuxi People’s Hospital affiliated to Nanjing Medical University, 299 Qingyang Road, Wuxi City, Jiangsu Province 214023, China

**Keywords:** Bcl-2, Breast cancer, Chemotherapy, Response

## Abstract

**Background:**

Numerous studies have yielded inconclusive results regarding the relationship between anti-apoptotic protein Bcl-2 expression and the sensitivity to chemotherapy in the patients with breast cancer. The purpose of the current study was therefore to elaborate their relationship.

**Methods, findings:**

A total of 23 previously published eligible studies involving 2,467 cases were identified and included in this meta-analysis. Negative Bcl-2 expression was associated with good chemotherapy response in breast cancer patients (total objective response [OR]: risk ratio [RR] = 1.16, 95% confidence interval [CI] = 1.02-1.32, p = 0.026; total complete response [CR]: RR = 1.67, 95% CI = 1.24-2.24, p = 0.001; pathological CR: RR = 1.92, 95% CI = 1.38-2.69, p < 0.001). In further stratified analyses, this association remained for sub-groups of response in neoadjuvant chemotherapy setting, especially pathological CR. Besides, negative Bcl-2 expression was significantly associated with good OR and pathological CR in anthracycline-based chemotherapy subgroup. Furthermore, there were significant links between negative Bcl-2 expression and taxane-based chemotherapy with pathological CR, but not OR.

**Conclusion:**

The results of the present meta-analysis suggest that Bcl-2 expression is a predictive factor for chemotherapy sensitivity in breast cancer patients. They could also potentially benefit further clinical treatment for breast cancers.

## Introduction

Breast cancer remains a major medical problem in women despite of dramatic advances in clinical and research have been achieved in the last three decades. Chemotherapies including neoadjuvant chemotherapy, adjuvant chemotherapy, and systemic chemotherapy, are widely used in breast cancer treatment. However, whether a patient responds to chemotherapy remains unpredictable, a proportion of patients fail to respond to chemotherapy, or even progress during therapy. Because the information on the drug sensitivity of tumors is often unknown before treatment initiation, many patients are treated, but only a few are benefited. Thus, predicting how well a patient will respond to chemotherapy and the risk of relapse is essential in deciding the best treatment option for each individual patient. Multiple biomarkers with potential predictive value have been evaluated in breast cancer, which may be useful for identifying those patients who would benefit from certain chemotherapy [1].

Conventional chemotherapeutic agents generally kill via the mitochondrial apoptotic pathway [2]. Mitochondrial priming is controlled by the Bcl-2 family of proteins [2,3]. This family consists of both pro-apoptotic and anti-apoptotic members. If pro-apoptotic members overwhelm the anti-apoptotic members, the threshold of death is crossed and the cell dies through apoptosis. The ability of Bcl-2 to prevent apoptosis is antagonized by the pro-apoptotic members of the Bcl-2 family [4]. Cytotoxic chemo-agents that promote apoptosis through DNA damage or microtubule disruption can be inhibited by Bcl-2 expression [5]. An *in vitro* study showed that over-expression of Bcl-2 increased the resistance of MCF-7 cells to doxorubicin, and this resistance was positively correlated with Bcl-2expression level of individual MCF/ Bcl-2 clones [6]. Studies demonstrated that Bcl-2 inhibition through targeted-RNAi knockdown or Bcl-2 antagonist (ABT-737) increased cellular response to daunorubicin, etoposide, and mitoxantrone in the THP-1 and OCI-AML3 cell lines [7], and targeting of the proteins Bcl-2 and Bcl-xL with ABT-737 may reverse the acquired radioresistance of MDA-MB-231R cells in vitro and in vivo [8].

Although there are now a large number of studies focusing on Bcl-2 expression in breast cancers, however, the association between its expression and chemosensitivity was not conclusive, mostly due to the small sample size of each study. We therefore performed a meta-analysis of the value of Bcl-2 expression for predicting sensitivity to chemotherapy in breast cancer.

## Materials and methods

### Publication search

PubMed, Embase, and Web of Science databases were searched (up to September 20, 2013) using the search terms: 'Bcl-2’, 'BCL2’, 'bcl’, 'bcl*’, 'B-cell CLL/lymphoma 2’, 'chemotherapy’ and 'breast cancer’. All potentially eligible studies were retrieved and their bibliographies were carefully scanned to identify other eligible studies. Additional studies were identified by a hand search of the references cited in the original studies. When multiple studies of the same patient population were identified, we included the published report with the largest sample size. Only studies published in English were included in this meta-analysis.

### Inclusion and exclusion criteria

Studies included in this meta-analysis had to meet all of the following criteria: (a) evaluation of Bcl-2 expression for predicting the response to chemotherapy in breast cancer, (b) studies with data on initial treatment, excluding studies reporting relapsed disease or second line therapy, (c) described therapeutic response, (d) retrospective or prospective cohort study, (e) inclusion of sufficient data to allow the estimation of a risk ratio (RR) with 95% confidence intervals (95% CI), and (f) studies published in English. Letters to the editor, reviews, and articles published in books, or papers published in a language other than English were excluded.

### Data extraction and definitions

According to the inclusion criteria listed above, the following data were extracted for each study: the first author’s surname, publication year, country of origin, number of patients analyzed, types of measurement, and the treatment. Data on the main outcomes were entered in tables showing the response to chemotherapy with respect to Bcl-2 expression. Information was carefully and independently extracted from all eligible publications by two of the authors (Yang and Chen). Any disagreement between the researchers was resolved by discussions until a consensus was reached. If they failed to reach a consensus, a third investigator (Lu) was consulted to resolve the dispute.

Response was defined as complete response (CR), partial response (PR), or objective response (OR) (OR = CR + PR). Non-response was defined as stable disease (SD) or progressive disease (PD), according to WHO criteria [9] or RECIST (Response Evaluation Criteria in Solid Tumors) criteria [10].

### Statistical analysis

RR with 95% CIs was used to estimate the association between Bcl-2 expression and response to chemotherapy in breast cancer patients. Subgroup analyses were performed to evaluate the effects of neoadjuvant chemotherapy and different treatment regimens (anthracycline-based and taxane-based). Heterogeneity assumption was checked using the Q test, and a p value >0.10 indicated a lack of heterogeneity among studies. We also quantified the effect of heterogeneity using *I*^2^ = 100% × (Q - df)/Q. *I*^2^ values of <25% may be considered "low", values of about 50% may be considered "moderate" and values of >75% maybe considered "high" [11]. In the absence of statistical heterogeneity, a fixed effects model was employed (the Mantel–Haenszel method). If heterogeneity was present, a random effects model (DerSimonian–Laird method) was used to account for inter-study heterogeneity. Funnel plots and the Egger’s test were employed to estimate the possible publication bias. We also performed sensitivity analysis by omitting each study or specific studies to find potential outliers. Statistical analyses were conducted using Stata (version SE/10; StataCorp, College Station, TX). p values for all comparisons were two-tailed and statistical significance was defined as p < 0.05 for all tests, except those for heterogeneity.

## Results

### Eligible studies

**Figure 1:**
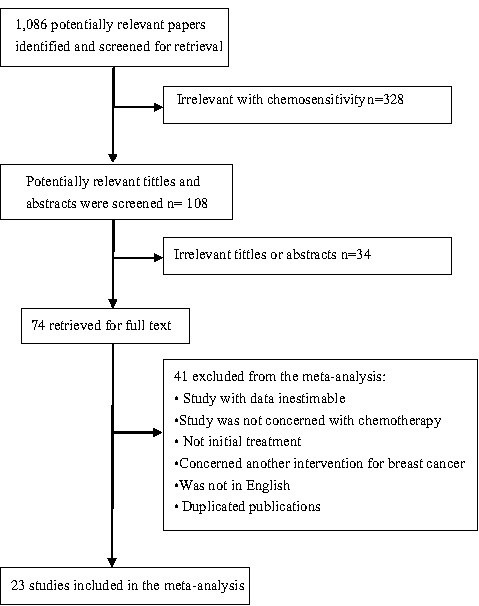
Improving the quality of reports of meta-analyses of randomized controlled trials; the Quality of Reporting of Meta-Analyses (QUOROM) statement flow diagram.

A total of 1,086 articles were retrieved by a literature search of the PubMed, Embase, and Web of Science databases, using different combinations of key terms. As indicated in the search flow diagram (Figure 1), 23 studies reported at least one of the outcomes of interest and were included in this meta-analysis [12-34]. The main features of these eligible studies are summarized in Table 1. The sample sizes in all the eligible studies ranged from 28–517 patients (median = 90 patients, mean = 107 patients, standard deviation [SD] =107). Overall, the eligible studies included a total of 2,467 patients. Nineteen of the studies were conducted in European or North American populations with mixed but mostly white participants (2,105 patients), whereas four were conducted in East Asian populations (362 patients).Data related to patients treated by neoadjuvant chemotherapy comprised 19 of the 23 breast cancer trials. Immunohistochemistry (IHC) techniques were used in all the trials to detect the expression of Bcl-2 protein. Various antibodies were used to assess Bcl-2 expression, and the cutoff in the number of positive cells defining a tumor with Bcl-2 overexpression varies from 5% to 50%, more than 10% for most studies (Table 1).

**Table 1:** Characteristics of studies included in the meta-analysis

**Author**	**Year**	**Country**	**Case**	**Disease stage**	**Method**	**Treatment**	**Detection**	**Provided information on cutoff value**	**Response**
Grim [12]	2012	Czech Republic	61	locally advanced	NCT	TAC	IHC	≥10% stained cell	Pathological CR
Chen [13]	2012	China	91	II-IIIC	NCT	PCb	IHC	≥10% stained cell	Pathological CR
Petrarca [14]	2011	Brazil	45	II-III	NCT	AC-T	IHC	>5(expression score range, 0–15)	Pathological CR
von Minckwitz [15]	2008	Germany	196	T_2-3_(≥3 cm) N_0-2_ M0	NCT	ddAT with or without tamoxifen	IHC	>1 + (range 0-3+)	Pathological CR
Vargas-Roig [16]	2008	Argentina	110	T_2–4_ N_0–1_ M0	NCT	FAC/FEC or D/E	IHC	>33% of stained cells	PR + CR
Keam [17]	2007	Korea,	145	II-III	NCT	docetaxel + doxorubicin	IHC	≥10% stained cell	OR
Tiezzi [18]	2006	Brazil	44	locally advanced	NCT	FEC or DE	IHC	≥5(range 0–7)	OR
Noguchi [19]	2006	Japan	63	NR	NR	Docetaxel	IHC	NR	OR
Mieog [20]	2006	Netherlands	107	T_1-4_	NCT	FEC	IHC	staining ≥3 indicates positive status	Pathological CR + OR
Fernandez-Sanchez [21]	2006	Mexico	40	IIB-IIIB	NCT	FAC	IHC	≥10% stained cell	OR
Prisack [22]	2005	Germany	517	locally advanced	NCT	EC ± RT	IHC	score ≥100	Pathological CR
Kim [23]	2005	Japan	63	tumor >3 cm and axillary lymph node involvement	NCT	docetaxel	IHC	Grades 2 and 3	Pathologic responders
Buchholz [24]	2005	USA	82	II-IV	NCT	FAC	IHC	presence of any cytoplasmic staining of the tumor cell cytoplasm	Pathological CR
Pusztai [25]	2004	USA	28	IIA-IIIB	NCT	FAC	IHC	any signal in neoplastic cells	Pathological CR
Ogston [26]	2004	UK	104	large and locally advanced	NCT	anthracycline-based ± docetaxel	IHC	≥10% stained cell	Good pathological response
Mathieu [27]	2004	France	129	T_2_ > 3 cm–T_4_	NCT	AVCMF or FAC/FEC	IHC	≥10% stained cell	Pathological CR
Stearns [28]	2003	USA	29	T_3_ or T_4_	NCT	A-T	IHC	Cytoplasmic staining.Intensity and % positive cells.Score ≥6 = positive	Pathological CR
Geisler [29]	2001	Norway	94	T_3_/T_4_ and/or N_2_ tumors	NCT and first-line	EC	IHC	index ≥ 6	PR
Pernick [30]	2000	USA	34	IIB or III	NCT	adriamycin (n = 32), taxol (n = 7), or taxotere (n = 7)	IHC	5% or more tumor cells stained.	CR
Bottini [31]	2000	Italy	157	T_2-4_ N_0-1_ M0	NCT	CMF ± tamoxifen or epirubicin	IHC	≥20% stained cell	CR + OR
Nole [32]	1999	Italy	39	T_1_-T_3_	NCT	FLN	IHC	>10% stained cell	OR
Colleoni [33]	1999	Italy	73	T_2_-T_3_,N_0-2_	NCT	FLN or AC	IHC	>10% stained cell	OR
Makris [34]	1997	UK	90	T_1_(N = 23),T_2_(n = 58),T_3_/T_4_(N = 9)	NCT	mitozantrone, methotrexate and tamoxifen	IHC	NR	OR

NCT, neoadjuvant chemotherapy; IHC, immunohistochemistry; A, doxorubicin; E, epirubicin; D, docetaxel; P, paclitaxel; F, 5-fluorouracil; EC, epirubicin and cyclophosphamide; FEC, 5-fluorouracil, epirubicin, and cyclophosphamide; FAC, 5-fluorouracil, doxorubicin, and cyclophosphamide; CMF, cyclophosphamide, mitomycin C and 5-fluorouracil; AVCMF, doxorubicin, vincristine, cyclophosphamide, methotrexate and 5-fluorouracil; P-FEC, sequential paclitaxel and 5-FU/epirubicin/cyclophosphamide; FUMI regimen, 5-fluorouracil (1,000 mg/m^2^ on days 1 and 2) and mitomycin; PCb, paclitaxel + carboplatin; RT radiation therapy; ddAT, dose-dense (bi-weekly) doxorubicin and docetaxel; FLN, 5-fluorouracil + folinic acid + vinorelbine; D and MF docetaxel; docetaxel to sequential methotrexate and 5-fluorouracil;NR, not reported.

### Evidence synthesis

**Figure 2:**
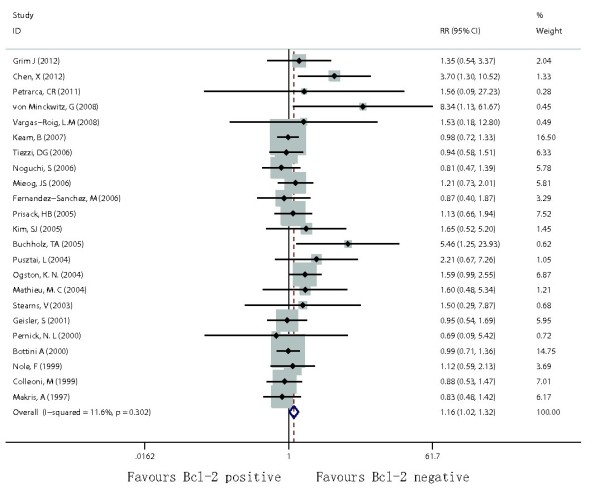
Forest plots of RR were assessed for association between Bcl-2 and total OR among breast cancer patients treated with chemotherapy.

Among the studies dealing with breast cancer patients with chemotherapy response, twenty-three studies involving 2,467 patients contributed to data on total OR (clinical OR + pathological OR). Negative Bcl-2 expression was significantly associated with improved total OR among patients with chemotherapy (RR = 1.16; 95% CI = 1.02–1.32; p = 0.026, Figure 2). Twelve studies involving 1,602 patients contributed to data on CR (pathological CR + clinical CR). Negative Bcl-2 expression was significantly associated with improved CR (RR = 1.67; 95% CI = 1.24–2.24; p = 0.001). Ten studies involving 1,285 patients contributed to data on total pathological CR. In fact, all these patients received neoadjuvant chemotherapy. Negative Bcl-2 expression was significantly associated with improved pathological CR (RR = 1.92; 95% CI = 1.38–2.69; p < 0.001).

### Subgroup analysis

**Figure 3:**
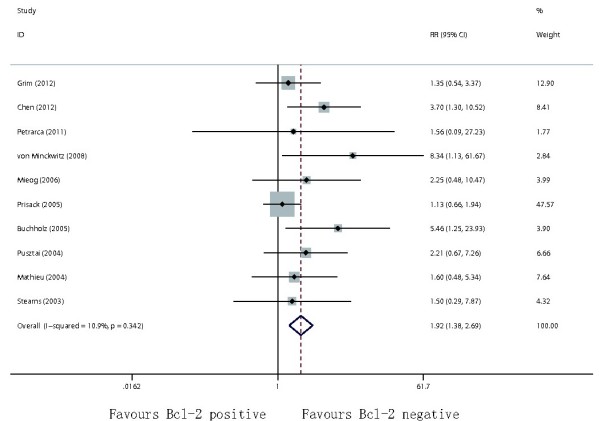
Forest plots of RR were assessed for association between Bcl-2 and pathological CR among breast cancer patients treated with neoadjuvant therapy.

Among the 23 studies, nineteen used neoadjuvant chemotherapy, one used first-line chemotherapy, one involving neoadjuvant and first-line chemotherapies, and one failed to report, we therefore calculated the associations with favorable responses to neoadjuvant chemotherapy. The results of subgroup analysis were presented in Table 2. Negative Bcl-2 expression was significantly associated with increased total OR (RR = 1.19, 95% CI = 1.04–1.37, p = 0.014), CR (RR = 1.67; 95% CI = 1.24–2.24; p = 0.001), pathological CR (RR = 1.92; 95% CI = 1.38–2.69; p < 0.001, Figure 3) among patients treated with neoadjuvant chemotherapy.

**Table 2:** Risk ratio for the association between Bcl-2 expression and response to chemotherapy

**Analysis**	**N**	**References**			**Heterogeneity**	
			**RR (95% CI)**	***P***	***I***^**2 **^**(%)**	***Ph***
All studies						
OR	23	[12-34]	1.16(1.02–1.32)	0.026*	11.6	0.502
CR	12	[12-15,20,22,24,25,27,28,30], [31]	1.67(1.24–2.24)	0.001*	15.8	0.289
Pathological CR	10	[12-15,20,22,24,25,27,28]	1.92(1.38–2.69)	<0.001*	10.9	0.342
Response to NCT						
OR	19	[12-18,20-22,24-26,28,30-34]	1.19(1.04–1.37)	0.014*	17.7	0.234
CR	12	[12-15,20,22,24,25,27,28,30], [31]	1.67(1.24–2.24)	0.001*	15.8	0.289
Pathological CR	10	[12-15,20,22,24,25,27,28]	1.92(1.38–2.69)	<0.001*	10.9	0.342
Response to anthracycline-based						
OR	15	[12,14-22,24-29]	1.20(1.01–1.43)	0.034*	15.5	0.280
Pathological CR	9	[12,14,15,20,22,24,25,27], [28]	1.76(1.24-2.51)	0.002*	0.0	0.452
Response to docetaxel-based						
OR	8	[12-15,17,19,23,28]	1.37(0.88–2.14)	0.160^#^	46.3	0.071
Pathological CR	4	[12-14,28]	2.11(1.14–3.88)	0.017*	0.0	0.525

N, number of studies; *Ph*, p value of Q-test for heterogeneity.

*The pooled RR was calculated using a fixed-effects model (the Mantel–Haenszel method) according to the heterogeneity.

#The pooled RR was calculated using a random-effects model (the DerSimonian and Laird method) according to the heterogeneity.

Subgroup analysis was performed when there were at least two studies in each subgroup.

**Figure 4:**
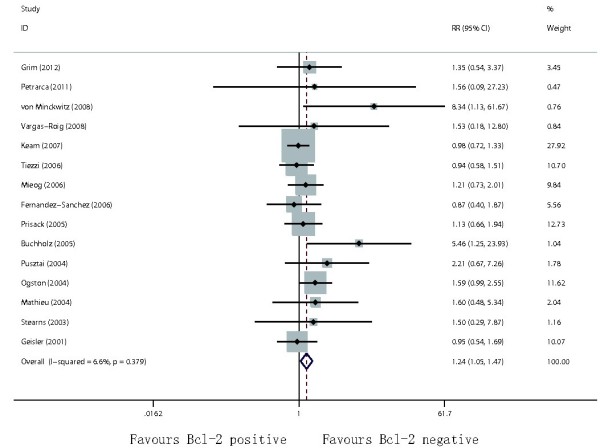
Forest plots of RR were assessed for the evaluation of total OR in anthracycline-based settings.

Bcl-2 expression has been used to evaluate associations with favorable responses to different treatment regimens of chemotherapy(either by anthracycline- or taxane-based). Among the 23 studies in the chemotherapy subgroup, fifteen used anthracycline-based neoadjuvant chemotherapy and eight used taxane-based neoadjuvant chemotherapy, while five used both anthracyclines and taxanes (Table 2). The results of the anthracycline- and taxane-based neoadjuvant chemotherapies were therefore calculated separately. No study contributed to data on clinical CR in the subgroup analysis. Negative Bcl-2 expression was associated with improved chemo-response in breast cancer patients who received anthracycline-based therapy (total OR: RR = 1.28, 95% CI = 1.01–1.43, p = 0.034, Figure 4; pathological CR: RR = 1.76, 95% CI = 1.24–2.51, p = 0.002). Negative Bcl-2 expression was significantly associated with increased pathological CR (RR = 2.11; 95% CI = 1.14–3.88; p = 0.017) among patients treated with taxane-based therapy, but not with total OR (RR = 1.37; 95% CI =0.88–2.14; p = 0.160).

### Publication bias

**Figure 5:**
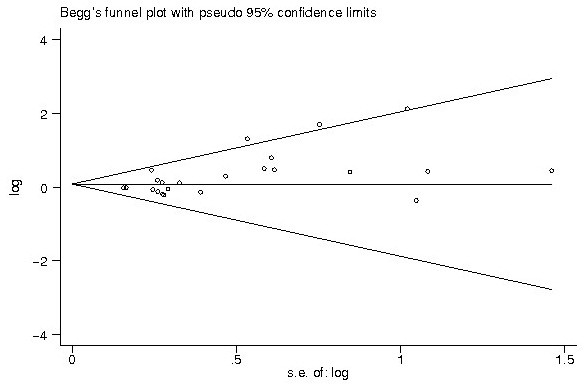
The funnel plot shows that there was no obvious indication of publication bias for the outcome of total OR.

**Figure 6:**
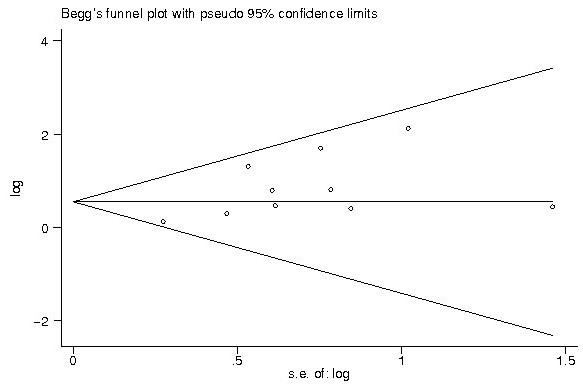
The funnel plot shows that there was no obvious indication of publication bias for the outcome of pathological CR among breast cancer patients treated with neoadjuvant therapy.

Begg’s funnel plot and Egger’s test were used to estimate the publication bias of the included literatures. The shapes of the funnel plots showed no evidence of obvious asymmetry (Figures 5 and 6), and Egger’s test indicated the absence of publication bias (p > 0.05). Moreover, sensitivity analysis was carried out to assess the influence of individual study on the summary effects. No individual study dominated this meta-analysis, and the removal of any single study had no significant effect on the overall conclusion (data not shown).

## Discussion

Although there have been many attempts to correlate Bcl-2 status with chemosensitivity in breast cancer patients, the results have been controversial. Our systematic review of the literatures shows that negative Bcl-2 expression is a good prognostic factor for predicting sensitivity to chemotherapy of breast cancers. The analysis reveals similar features in different subgroups and clarifies the message of individual studies that are somewhat inconsistent.

In our systematic review with meta-analysis, patients with Bcl-2-negative tumors had significantly better response to chemotherapy than those with Bcl-2-positive tumors. This observation is potentially important. The identification of independent predictive factor allows us to define high-risk patients for whom specific therapy may be designed or to introduce stratification in randomized trials. Furthermore, identifying and validating a predictive biomarker is of more than purely scientific interest because clinical Bcl-2 inhibition is now a practicable clinical approach. Recently, Abbott Laboratories has two drugs in clinical trials that directly target Bcl-2, ABT-263, and ABT-199 [35-37].

Interestingly, we noticed that patients with Bcl-2-negative tumors were more likely to achieve a complete remission after neoadjuvant chemotherapy. Multiple clinical trials have shown that patients who achieved a pathological CR after neoadjuvant chemotherapy were associated with improved survival. Intensive researches have thus been focused on identification of “predictive markers” of chemotherapy sensitivity, especially those producing pathological CR [38]. Our results indicated that Bcl-2 expression could potentially help to stratify patients in neoadjuvant chemotherapy.

Notably, we found the correlation between Bcl-2 expression and the response to anthracycline-based chemotherapy. It is important to mention that Vo TT *et al*. found that Bcl-2 MOLM13 partial knockdown was associated with increased death by topoisomerase II inhibitors (etoposide, daunorubicin, and mitoxantrone) in proportion to the quality of the knockdown [7].

A combination of the analysis of Bcl-2 expression and perhaps other variables (e.g., HER-2, hormone receptor, tumor size and histological subtype) may make it possible, to stratify chemotherapy sensitive subgroup of patients with advanced breast cancer.

The decision to perform the meta-analysis was based on a prior methodological assessment of the publications. We have used a methodology similar to previous systematic reviews reported by our group on the treatment of breast cancer [8] and rectal cancer after an adaptation to biological predictive factors such as p53 [39]. By comparing the scores of the studies where Bcl-2 was a significant predictive factor and those where it was not, we could identify differences, suggesting biases induced by trial methodology. Nevertheless, our approach does not eliminate all potential biases. First, the meta-analysis may have been influenced by publication bias, we limited the search to studies written in English, and we did not search conference proceedings and abstract books, which may have introduced publication bias to this meta-analysis. We tried to identify all relevant data and retrieve additional unpublished information, some missing data were, however, unavoidable. Second, the techniques used to identify overexpression of Bcl-2 status can also be a potential source of bias. The IHC used to reveal the Bcl-2 protein is not always performed with the same antibody. Moreover, the cutoff in the number of positive cells defining a tumour with Bcl-2 overexpression often varies according to the investigators, which may lead to biased conclusions. Third, although we made considerable efforts to standardize definitions, some variability in definitions of methods, measurements, and outcomes among studies was inevitable . Fourth, our analysis was observational in nature, and we therefore can’t exclude confounding as a potential explanation of the observed results. Despite these limitations, this meta-analysis had several strengths. First, a substantial number of cases were pooled from different studies, and 2,467 subjects represents a sizeable number to significantly increase the statistical power of the analysis. Secondly, no publication biases were detected, indicating that the pooled results may be unbiased.

This study is the first meta-analysis to assess the use of Bcl-2 expression for predicting the chemo-sensitivity of breast cancer patients. Our data support Bcl-2 expression as a useful predictive factor for assessing treatment response to chemotherapy in breast cancer patients. However, future properly designed prospective studies with large sample sizes and an appropriate statistical methodology including multivariate analysis are required to confirm our findings. Moreover, the interaction of this marker with other markers such as HER-2, hormone receptor, tumor size and histological subtype remains unknown and should be a matter for further investigation.

## Abbreviations

CI: Confidence interval; IHC: Immunohistochemistry; OS: Overall survival; RR: Relative risk; OR: Objective response; CR: Complete response.

## Competing interests

The authors declare that they have no competing interests.

## Authors’ contributions

DY, M-BC, P-HL contributed to the conception and design of the study, the analysis and interpretation of data, the revision of the article as well as final approval of the version to be submitted. LY, C-YL and L-QW. participated in the design of the study, performed the statistical analysis, searched and selected the trials, drafted and revised the article. All authors read and approved the final version of the manuscript.
